# Commentary: Noninvasive assessment of anomalous aortic origin of the right coronary artery: A simulated reality?

**DOI:** 10.1016/j.xjtc.2022.04.003

**Published:** 2022-04-16

**Authors:** Gregory King, Igor E. Konstantinov

**Affiliations:** Department of Cardiac Surgery, The Royal Children's Hospital, Melbourne, Australia; Heart Research, Murdoch Children's Research Institute, Melbourne, Australia

**Figure:**
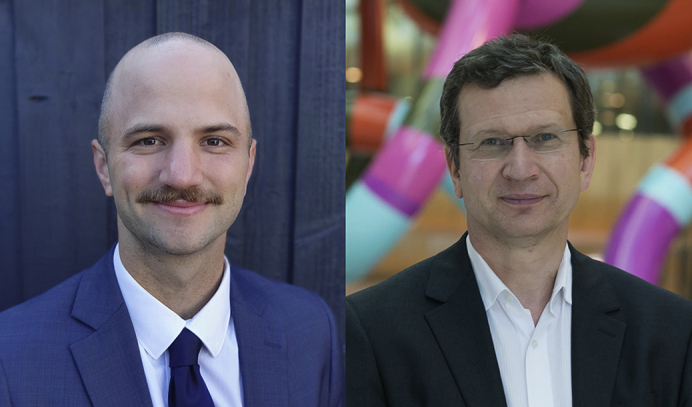
Gregory King, MD, and Igor E. Konstantinov, MD, PhD, FRACS

**Central Message**

CTA-based FSI modeling holds promise as an adjunctive tool in the assessment of ischemia risk in patients with AAORCA; however, this remains an unresolved and incompletely understood problem.

See Article page 144.

**Figure 1:**
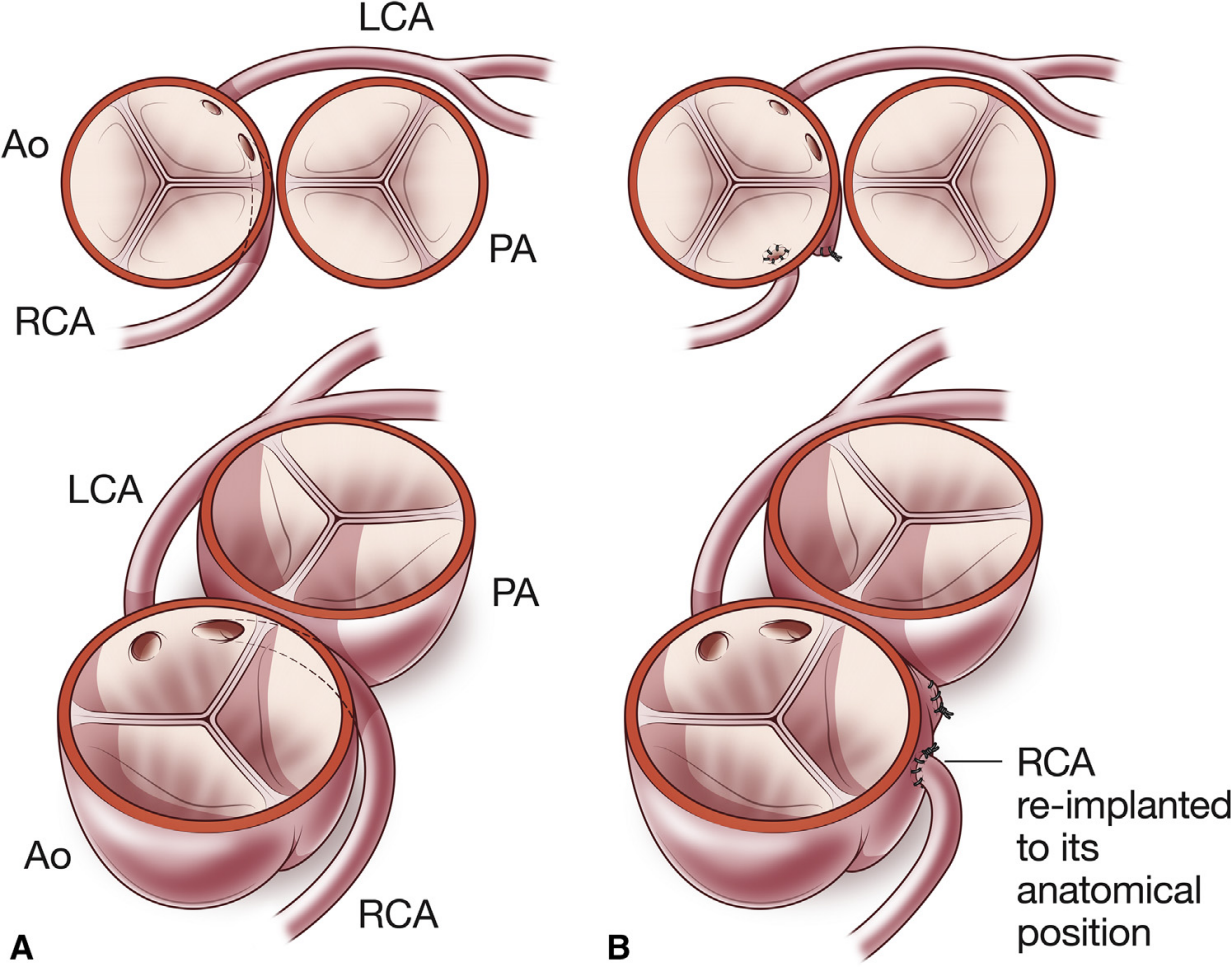
An anomalous right coronary artery (*RCA*) arising from the left sinus of Valsalva (A) reimplanted into its anatomical position (B). *Ao*, Aorta; *LCA*, left coronary artery; *PA*, pulmonary artery.

Anomalous aortic origin of a coronary artery is the second-leading cause of death in otherwise-healthy youth.^1^ It is clear that a symptomatic patient with an anomalous right coronary artery (RCA) arising from the aorta (ie, anomalous aortic origin of the right coronary artery [AAORCA]) should undergo surgical correction.^2^, ^3^, ^4^ However, it is not clear what to do with an asymptomatic individual. Although the operation to reimplant the RCA is relatively straightforward (Figure 1), it is not without risk,^5^^,^^6^ and the optimal management of an asymptomatic person with AAORCA is yet to be defined.^4^ In this issue of the *Journal*, Jiang and colleagues^7^ described the use of computed tomography angiogram (CTA)-based fluid–structure interaction (FSI) modeling as an adjunctive tool for assessing both the burden and mechanism of potential ischemia in patients with AAORCA. In doing so, they have provided an important contribution toward risk stratification of asymptomatic patients with AAORCA.

The study included 6 patients with AAORCA and atypical cardiac symptoms, all of whom had an interarterial course and all but 1 patient had an intramural course of the RCA. Their modeling demonstrated that the intramural segment of the lumen is restricted from expanding during exercise, thereby acting as a “functional stenosis” and leading to a greater pressure drop across the intramural segment of the artery. A sensitivity analysis was performed, omitting the effect of the pulmonary root from modeling (albeit only in 1 patient), and the simulated instantaneous wave-free ratio (iFR) did not significantly differ as a result. As such, in patients with AAORCA, the mechanism of ischemia appears to be mainly due to the intramural course of the artery, rather than the interarterial course and compression from the adjacent pulmonary artery.

In addition to providing insight into the mechanism of ischemia, CTA-based FSI modeling also appears capable of accurately assessing the burden of ischemia in patients with AAORCA. More specifically, there was good correlation between rest and dobutamine stress iFR obtained from the reference standard of invasive measurement and those derived from CTA-based FSI modeling. However, it is notable that within a small cohort, 1 patient had an invasive iFR of 0.95, but a simulated iFR of 0.86, which would have led to discordant recommendations regarding surgical intervention.

Ultimately, this sophisticated study by Jiang and colleagues^7^ provides a very important contribution to the literature. Needless to say, the study only included 6 patients and before being adopted for clinical practice, CTA-based FSI modeling will need to be trialed among a larger cohort to assess its applicability to a broader range of patients. Although an intramural course appears to be the primary determinant of ischemia in AAORCA, the exact mechanisms of ischemia remain incompletely understood. Furthermore, the decision of whether to intervene or monitor asymptomatic individuals with AAORCA remains ambiguous. Part of this dilemma relates to the difficulty establishing the true risk of sudden cardiac death in patients with AAORCA, and therefore it is difficult to balance the risk of surgical repair against the risk of continued surveillance.^8^^,^^9^ Will an intramural course of the RCA by itself will become an indication for RCA reimplantation? This is yet to be determined.
